# Chronic Obstructive Pulmonary Disease (COPD) in Population Studies in Russia and Norway: Comparison of Prevalence, Awareness and Management

**DOI:** 10.2147/COPD.S292472

**Published:** 2021-05-14

**Authors:** Sarah Cook, Anne Elise Eggen, Laila A Hopstock, Sofia Malyutina, Marina Shapkina, Alexander V Kudryavtsev, Hasse Melbye, Jennifer K Quint

**Affiliations:** 1 Department of Community Medicine, UiT The Arctic University of Norway, Tromsø, Norway; 2 Faculty of Epidemiology and Population Health, London School of Hygiene & Tropical Medicine, London, UK; 3 National Heart and Lung Institute, Imperial College London, London, UK; 4 Research Institute of Internal and Preventive Medicine, Branch of Institute of Cytology and Genetics, Siberian Branch of the Russian Academy of Sciences, Novosibirsk, Russian Federation; 5 Novosibirsk State Medical University, Russian Ministry of Health, Novosibirsk, Russian Federation; 6 Northern State Medical University, Arkhangelsk, Russian Federation

**Keywords:** COPD, Russian Federation, Norway, respiratory symptoms

## Abstract

**Background:**

Chronic obstructive pulmonary disease (COPD) is a major cause of morbidity and mortality worldwide. Despite a high prevalence of smoking and respiratory symptoms, two recent population-based studies in Russia found a relatively low prevalence of obstructive lung function. Here, we investigated the prevalence of both obstructive lung disease and respiratory symptoms in a population-based study conducted in two Russian cities and compared the findings with a similar study from Norway conducted in the same time period.

**Methods:**

The study population was a sub-sample of participants aged 40–69 years participating in the Know Your Heart (KYH) study in Russia in 2015–18 (n=1883) and in the 7th survey of the Tromsø Study (n=5271) carried out in Norway in 2015–16 (Tromsø 7) who participated in spirometry examinations. The main outcome was obstructive lung function (FEV_1_/FVC ratio< lower limit of normal on pre-bronchodilator spirometry examination) with and without respiratory symptoms (chronic cough and breathlessness). In those with obstructive lung function, awareness (known diagnosis) and management (use of medications, smoking cessation) were compared.

**Results:**

The age-standardized prevalence of obstructive lung function was similar among men in both studies (KYH 11.0% vs Tromsø 7 9.8%, p=0.21) and higher in the Norwegian (9.4%) than Russian (6.8%) women (p=0.006). In contrast, the prevalence of obstructive lung function plus respiratory symptoms was higher in Russian men (KYH 8.3% vs Tromsø 7 4.7%, p<0.001) but similar in women (KYH 5.9% vs Tromsø 7 6.4%, p=0.18). There was a much higher prevalence of respiratory symptoms in Russian than Norwegian participants of both sexes regardless of presence of obstructive lung function.

**Conclusion:**

The prevalence of respiratory symptoms was strikingly high among Russian participants but this was not explained by a higher burden of obstructive lung function on spirometry testing in comparison with Norwegian participants. Further work is needed to understand the reasons and health implications of this high prevalence of cough and breathlessness.

## Introduction

Despite recent declines, premature mortality remains a major public health concern in Russia.^1^ Respiratory diseases contribute substantially with Chronic Obstructive Pulmonary Disease (COPD) estimated as the 14th leading cause of death in Russia in 2016 while respiratory infection was the 6th leading cause of death.^1^ COPD is strongly related to cardiovascular disease (CVD)^2^,^3^ and in many patients with COPD cardiovascular disease is the underlying cause of death.^4–6^ In Russia, there is a high prevalence of smoking in men,^7^ which is a major risk factor for COPD. Occupational exposure to vapours, dust and fumes may also lead to increased risk among certain groups.^8^ It is important to quantify and understand the burden of COPD within Russia, particularly in a country with one of the highest rates of CVD mortality in the world.^9^

Four population-based surveys have found high levels of reporting of respiratory symptoms in the Russian general population^10–13^ but only two of these studies also included spirometry testing.^11^,^13^ In a cross-sectional survey of 7164 adults aged 18 or older living in 12 regions of Russia (2010–2011), spirometry was used to diagnose COPD among those who reported any respiratory symptoms or risk factors for chronic respiratory disease^11^. Chuchalin et al^11^ extrapolated results from spirometry testing in this study to estimate that the prevalence of symptomatic COPD (symptoms plus forced expiratory volume in 1 second (FEV_1_): Forced vital capacity (FVC) ratio<0.7) in the total study population was 15.3%. Estimates of airway obstruction from the earlier HAPIEE study (2002–2005) from 6875 men and women aged 45–69 years old in the city of Novosibirsk were even higher at 19.5% (23.5% in men and 16.0% in women) using a broader definition of airway obstruction on spirometry (FEV_1_/FVC ratio <0.7 or FEV_1,_<80%).^14^ However, a study of 2975 adults aged 35–70 living in North West Russia (2012–13) found a substantially lower prevalence of airway obstruction of 6.8% using a fixed FEV_1_:FVC ratio<0.7 and 4.8% using the Global Lung Initiative Lower Limit of Normal (GLI-LLN) cut off with a substantial sex difference (higher in men (9.6%) than women (4.8%)).^13^ This was consistent with estimates from pre-bronchodilator spirometry from a later study of 5899 adults aged 40–94 in the Russian region of Bashkortostan which found a prevalence of airway obstruction using LLN of 5.8% (6.8% using fixed cut point).^15^ These prevalence estimates are lower than might be expected given the high burden of smoking^7^ and self-reported respiratory symptoms^10–13^ in the Russian general population.

In quantifying the prevalence and relative disease burden from COPD in Russia findings need to be compared in context with studies from other populations. However, making comparisons between population-based studies is complex. Estimated prevalence of COPD can vary widely depending on the criteria used for case definition.^16^,^17^ Prevalence of COPD in Norway, a neighbouring country to Russia with a different mortality and risk factor profile, shows considerable variation. Estimates from the BOLD study site in Bergen (2006) which used spirometry only found the prevalence was 11% using postbronchodilator fixed cut point among adults aged 52–60.^18^ Findings from the population-based HUNT-3 study in the county of Nord-Trøndelag (2006–2008) found a prevalence of 14.5% using a fixed cut point and 7.3% from LLN in adults aged over 40 on pre-bronchodilator spirometric assessment.^19^ Analyses of changes in COPD prevalence over time within the Tromsø Study in the municipality of Tromsø have shown COPD prevalences have been declining since 2001 in line with declines in smoking, with the most recent estimates from 2015–2016 of 9.7% in men and 10.0% in women aged 40–84 using LLN and 5.6% in both sexes when including respiratory symptoms within the definition.^20^ However, to compare estimated COPD prevalence from Russia with studies from other settings is difficult given differences in age range, COPD definition and time frames.

Here we investigated prevalence of both obstructive lung disease and respiratory symptoms in a population-based study conducted in two Russian cities and compared the findings with a similar study from Norway conducted in the same time period using the same definitions of COPD for both studies. Among those with evidence of COPD, we compared levels of awareness and management (smoking cessation, use of pharmacological treatments) between the study populations.

## Methods

### Study Populations

The study population was a sub-sample of participants aged 40–69 years taking part in the two population-based health surveys the Know Your Heart (KYH) Study^21^ conducted in the Russian cities of Arkhangelsk and Novosibirsk (2015–18) and the seventh survey of the Tromsø Study^22^ (Tromsø 7) conducted in the Norwegian municipality of Tromsø (2015–16). These studies were conducted in parallel and several aspects of data collection between the studies have been harmonized (including the measurement of breathlessness, quality criteria for spirometry and ATC coding of medications) providing a unique opportunity to compare lung function between general population samples in both countries.

### Know Your Heart Study Sample (Russia)

Participants were identified from a population register of addresses and a random sample stratified by age, sex and district was selected (n=15,284 aged 40–69). Trained interviewers visited the addresses and invited participants to take part in the study. Participants who agreed to take part completed an interviewer-administered questionnaire which included questions on socio-demographic factors and self-reported morbidities (n=4654 aged 40–69). Participants were then invited to a health check which included a further questionnaire and a comprehensive medical examination (n= 4044 aged 40–69). Spirometry was an additional component of the health check offered to approximately 50% of participants. Selection of participants, for practical reasons, was determined by the day of the week that medical professionals trained in these procedures were available. The days of the week when spirometry was offered were varied throughout the fieldwork in order to minimize selection bias. Contra-indications for spirometry were: chest infection in the last month (ie, influenza, bronchitis, severe cold, pneumonia); history of detached retina; myocardial infarction in the past month; surgery to eyes, chest or abdomen in last 3 months; history of a collapsed lung; pregnancy (1st or 3rd trimester); currently on medications for tuberculosis.

Uptake was high among those to whom it was offered (94.9% of participants invited to spirometry completed the examination) and in total lung function data are available for 45.7% of participants who attended the health check (n=1883 aged 40–69). Data on use of medications, smoking and self-report of respiratory symptoms were collected for all participants attending the health check.

### The Tromsø Study Sample (Norway)

All inhabitants aged 40 and older were invited to take part in Tromsø 7 (n=32,591). Participants underwent a basic examination which involved questionnaires and interviews, biological sampling and clinical examinations (n=21,083 of which n=17,646 were aged 40–69). A subset of participants (randomly pre-marked before attendance with addition of previous participants from Tromsø 6 2007–2008) were invited to take part in extended examinations including spirometry. There were no contra-indications for spirometry assessment. In total spirometry was conducted on 5217 participants in the required age range.

### Spirometry Assessment

In KYH Spirometry was conducted using 6800 Pneumotrac spirometers (Vitalograph^®^, UK) and in Tromsø 7 using Vmax^®^ Encore devices (Sensormedics^®^ Corporation, USA). The spirometry examination took place alongside several other clinical examinations in both studies. Post-bronchodilator measurements were not taken in order to minimize burden for participants.

For both studies, three measurements were taken. If less than 2 of the measures were acceptable then additional measurements were taken up to a maximum of eight. Maximum FEV_1_ and maximum FVC were used in analyses not necessarily from the same blow. Following data collection acceptability and reproducibility of results were determined by 1) removing blows where FEV_1_ was less than 300 mL and 2) excluding values where FEV_3_ was the same or greater than FVC. For Tromsø 7 the curves from the study were assessed and cleaned manually by the study team responsible for data collection. For KYH cases where the difference between maximum values and the second highest value for FEV_1_ and FVC were greater than 250 mL were evaluated. In cases where the maximum value is found to be an outlier (difference between 2nd and 3rd max<250mL) compared to other values the next highest value was used. Otherwise, the maximum was used.

### Outcome

The primary outcome definition was pre-bronchodilator FEV_1_: FVC ratio<Lower Limit Normal (LLN) with and without self-reported respiratory symptoms. LLN was calculated from the GLI LLN equations^23^ specifying ethnicity as white using GLI-2012 Desktop Software for Large Data Sets.^24^ The secondary definition of FEV_1_: FVC ratio<0.7 with and without symptoms was used to investigate impact of definition of the findings.

Respiratory symptoms were defined as chronic cough and/or breathlessness. Breathlessness was measured in both studies using the MRC breathlessness scale.^25^ Breathlessness was categorised as grade 2 breathlessness or above (equivalent to grade 1 on the modified mMRC dyspnoea scale) “short of breath when hurrying on the level or walking up a slight hill”. Chronic cough was defined from the questions in Table 1. If participants answered “yes” to any of the questions on cough they were considered to have a chronic cough. Sensitivity analyses were conducted using a stricter definition of both breathlessness and cough to define respiratory symptoms.

**Table 1:** Questions on Chronic Cough

**Know Your Heart**	**Tromsø 7**
Do you usually cough first thing in the morning in winter?	Do you cough about daily for some periods of the year?
Do you usually cough during the day or at night in winter?	If you cough about daily for some periods of the year, is your cough productive?
Do you cough like this on most days for as much as three months each year?	If you cough about daily for some periods of the year, have you had this kind of cough for as long as 3 months in each of the last two years?
Do you usually bring up phlegm from your chest first thing in the morning in winter?	
Do you usually bring up any phlegm from your chest during the day- or at night – in winter?	
Do you bring up phlegm like this on most days for as much as three months each year?	

### Risk Factors

Risk factors measured were age, sex, education, smoking status (never, ex-smoker, current smoker) and pack-year history calculated from questions on years smoked and number of cigarettes smoked per day, and body mass index (BMI) calculated from measured height and weight. Education was coded into three categories (lower, middle and higher) based on the education system within each country. In KYH these groups were lower (incomplete secondary and vocational no secondary), middle (complete secondary, vocational and secondary, specialised secondary) and higher (incomplete higher, higher) education. For Tromsø 7, these were lower (primary) middle (upper secondary) and higher (university/university college) education. Current smokers in both studies included participants those who smoked less than daily (KYH n=26; Tromsø 7 n=350).

An indicator of existing CVD based on self-reported stroke and/or myocardial infarction was also included as an important factor associated with respiratory disease with different expected prevalence between the two studies.

### Awareness of COPD

Awareness was assessed from self-report of chronic lung disease. In KYH this was assessed with the question “Have you ever been told by a doctor (been diagnosed) that you have chronic bronchitis/COPD?” In Tromsø 7 this question was “Have you ever had, or do you have chronic bronchitis/emphysema/COPD?”. Participants who reported they had a diagnosis either now or previously were categorised as aware of COPD status.

### Management of COPD

Management of COPD was compared on two levels: smoking cessation and pharmacological management according to Global Initiative for Chronic Obstructive Disease (GOLD) guidelines.^26^

Levels of smoking cessation were assessed by comparing the proportion of those with obstructive lung function ± respiratory symptoms who were current smokers. In the KYH two additional questions on smoking cessation advice and assistance were asked to participants who reported they were smokers “Have you ever been advised by a medical professional (your GP, cardiologist, any other physician) to stop smoking?” and “was any assistance offered?”

For both studies, data on use of current medications were collected and coded in accordance with the International WHO Anatomical Therapeutic Chemical (ATC) classification system version 2016.^27^ Pharmacological treatment was compared across two domains of use applicable to participants in population-based studies: maintenance treatment and short-acting symptomatic treatment. Maintenance treatment was defined as the use of long-acting muscarinic antagonists (R03BB) (LAMA); long-acting beta-2 agonists (R03AC12, 13, 18 and 19) (LABA); combination of long-acting beta-2 agonists with steroids or long-acting muscarinics (R03AK, R03AL); theophylline (R03DA04); roflumilast (R03DX07)). Short-acting symptomatic treatment was defined as the use of short-acting beta agonists (R03AC02, 03, 04)).

### Statistical Analysis

The prevalence of obstructive lung function (plus symptoms) was compared by study and sex with 1) standardisation for age to the 2013 Standard European population and 2) stratification across 10 year age bands.

Differences in the studies in the associations with age, sex, education, smoking, BMI and self-reported CVD were investigated by fitting separate logistic regression models with the outcomes 1) obstructive lung function and 2) obstructive lung function plus symptoms and testing for interactions between study and each risk factors using likelihood ratio tests.

Interaction between age and pack-year history was investigated within each study by fitting logistic regression models with 1) obstructive lung function and 2) obstructive lung function plus symptoms as the outcomes adjusted for sex (and city for KYH) with and without interaction terms between age and pack-year history and comparing these using likelihood ratio tests. These interactions were investigated because of observed differences in the distribution of age and pack-year history between the studies.

Prevalence of current smoking, self-report of diagnosis, and medication use in those with obstructive lung disease was compared between the studies across levels of reported respiratory symptoms (none, one symptom or both symptoms). Differences between studies were investigated using separate logistic regression models for each outcome and study as the exposure adjusted for 1) age and sex and 2) age, sex and respiratory symptoms.

Interaction by sex and study was investigated using likelihood ratio tests and if there was evidence suggesting interaction results were presented stratified by sex.

### Sensitivity Analysis to Investigate the Impact of Missing Data

The main analyses were restricted to complete case analysis.

As spirometry was conducted in a sub-set of participants in both studies, the potential impact of this was investigated by comparing the characteristics of those with spirometry data to the full sample (all health check attendees for KYH, all basic examination attendees Tromsø 7).

To investigate whether differences in those included and excluded could have affected the estimates of prevalence of obstructive lung function (<LLN) sensitivity analyses were conducted. Separate multiple imputation models were fitted for each study imputing the outcome in those who did not complete the spirometry examination from age, sex, education, BMI, self-reported CVD, smoking status, pack-year history, chronic cough and MRC breathlessness scale.

## Results

In total there were 1883 participants in KYH (41.8% men) and 5217 participants in Tromsø 7 (45.7% men) aged 40–69 with data from spirometry. Characteristics of participants by sex and study are shown in Table 2. Although the proportion of men in the oldest age group was higher for the Tromsø 7 men, the KYH men had a higher smoking pack-year history.

**Table 2:** Characteristics of Participants by Sex and Study. Know Your Heart Study 2015–2018 and Tromsø Study 2015–2016

		Know Your Heart				Tromsø 7			
		Women		Men		Women		Men	
		N	(%)	N	(%)	N	(%)	N	(%)
	Total sample	1096	(100)	787	(100)	2831	(100)	2386	(100)
Age, years	40–49	331	(30.2)	238	(30.2)	553	(19.5)	471	(19.7)
	50–59	384	(35.0)	259	(32.9)	716	(25.3)	526	(22.1)
	60–69	381	(34.8)	290	(36.9)	1562	(55.2)	1389	(58.2)
City	Arkhangelsk	538	(49.1)	405	(51.5)	–		–	
	Novosibirsk	558	(50.9)	382	(48.5)	–		–	
Education	Lower	58	(5.3)	63	(8.0)	660	(23.6)	537	(22.7)
	Middle	586	(53.5)	405	(51.5)	782	(28.0)	717	(30.4)
	Higher	452	(41.2)	319	(40.5)	1356	(48.5)	1107	(48.9)
	Missing	0		0		33		25	
Smoking status	Never	744	(67.9)	217	(27.6)	958	(34.1)	856	(36.3)
	Ex-smoker	173	(15.8)	285	(36.3)	1453	(51.7)	1131	(47.9)
	Current smoker	179	(16.3)	283	(36.1)	400	(14.2)	374	(15.8)
	Missing	0		2		20		25	
Smoking pack years among ever smokers	>0<10	184	(60.1)	85	(15.5)	940	(52.6)	564	(38.2)
	10–19	54	(17.7)	104	(18.9)	478	(26.8)	433	(29.3)
	20–29	45	(14.7)	163	(29.6)	246	(13.8)	220	(14.9)
	30–39	14	(4.6)	109	(19.8)	83	(4.6)	167	(11.3)
	40+	9	(2.9)	89	(16.2)	40	(2.2)	92	(6.2)
	Missing	46		18		66		29	
Body mass index, kg/m^2^	<18.5	13	(1.2)	9	(1.2)	29	(1.0)	2	(0.1)
	18.5–24.9	317	(29.0)	218	(27.7)	1119	(39.6)	572	(24.0)
	25–29.9	361	(33.0)	349	(44.4)	1095	(38.7)	1208	(50.7)
	30–34.9	259	(23.7)	166	(21.1)	430	(15.2)	471	(19.8)
	>35	144	(13.2)	44	(5.6)	154	(5.5)	130	(5.5)
	Missing	2		1		4		3	
Self-reported Cardiovascular co-morbidity (stroke and/or myocardial infarction)	Yes	67	(6.3)	94	(12.3)	80	(3.0)	178	(7.8)
	Missing	24		21		127		98	
Chronic cough	Yes	455	(41.8)	335	(43.2)	397	(14.2)	412	(17.5)
	Missing	7		11		27		31	
Breathlessness grade 2	Yes	672	(62.2)	306	(39.3)	854	(30.2)	593	(24.9)
	Missing	15		8		7		8	
Respiratory symptoms	None	283	(26.4)	316	(41.0)	1740	(62.2)	1517	(64.6)
	Either cough or breathless	467	(43.5)	273	(35.5)	873	(31.2)	666	(28.4)
	Both cough and breathless	324	(30.2)	181	(23.5)	184	(6.6)	164	(7.0)
	Missing	22		17		34		39	
FEV_1_: FVC ratio<0.7	Yes	123	(11.2)	154	(19.6)	542	(19.2)	526	(22.1)
FEV_1:_ FVC ratio<LLN*	Yes	75	(6.8)	88	(11.2)	290	(10.2)	234	(9.8)
FEV_1:_ FVC ratio<0.7 plus symptoms**	Yes	104	(9.6)	113	(14.6)	257	(9.2)	237	(10.1)
	Missing	22		17		34		39	
FEV_1:_ FVC ratio<LLN* plus symptoms**	Yes	64	(5.9)	67	(8.6)	151	(5.4)	123	(5.2)
	Missing	22		17		34		39	
Ever diagnosed	Yes	216	(19.7)	108	(13.8)	96	(3.5)	78	(3.4)
	Missing	1		3		97		61	

**Notes:** *GLI LLN (z-score for FEV_1_/FVC ratio<-1.64). **Symptoms defined as cough and/or breathlessness.

There was no evidence for a difference in sex-stratified prevalences of obstructive lung function between the two Russian sites after adjusting for age, and due to small numbers findings from both sites were pooled.

### Prevalence of Obstructive Lung Function and Respiratory Symptoms

The crude prevalence of obstructive lung function and respiratory symptoms by sex and study is shown in Table 2. Findings after age-standardisation were similar to the crude findings. Among women the age-standardized prevalence of obstructive lung function defined by LLN was higher (age-adjusted p value=0.006) in the Tromsø 7 women (9.4%) compared to the KYH women (6.8%) while there was no evidence for a difference by study in men (KYH men 11.0%; Tromsø 7 men 9.8% age-adjusted p value=0.21).

**Figure 1:**
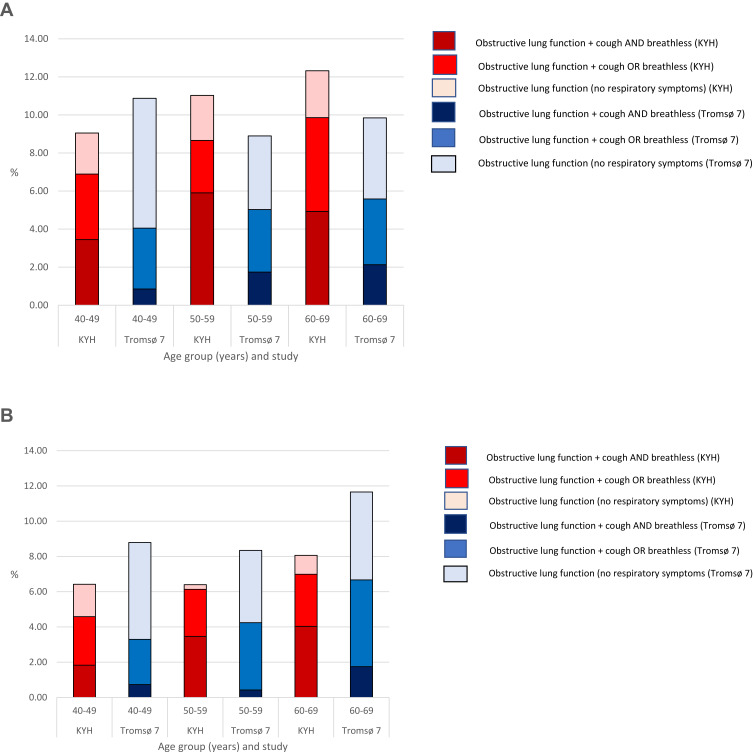
Prevalence of obstructive lung disease (FEV1: FVC Ratio<GLI LLN) with and without respiratory symptoms by age and study in men and women. (**A**) Prevalence of obstructive lung disease (FEV1: FVC Ratio<GLI LLN) with and without respiratory symptoms in men. (**B**) Prevalence of obstructive lung disease (FEV1: FVC Ratio<GLI LLN) with and without respiratory symptoms in women.

The prevalence of reporting respiratory symptoms among those with obstructive lung function was substantially higher in KYH than Tromsø 7 in both men and women across all ages (Figure 1A and B). The age-standardized prevalence of COPD defined as obstructive lung function and one or more respiratory symptom was 8.3% in KYH men and 4.7% in Tromsø 7 men (age-adjusted p value<0.001). In women, this was 5.9% in KYH and 4.6% in Tromsø 7 (age-adjusted p value=0.18).

The positive predictive value of any respiratory symptoms for identifying obstructive lung function was low in both studies (10.4% in KYH; 14.4% in Tromsø 7). The marked differences in reporting of respiratory symptoms between the studies were also seen in those without obstructive lung function on spirometry (Supplementary Figure 1).

### Interaction in the Between-Study Differences by Sex, Age, Education, Smoking, BMI and Self-Reported CVD

The associations between obstructive lung function and age, sex, education, smoking, BMI and self-reported CVD morbidity and interactions by study are shown in Table 3. There was no evidence for interaction in associations with age, education, smoking, BMI or self-reported CVD morbidity between the studies but there was strong evidence for a difference in association with sex (p=0.002). Women had lower odds of obstructive lung disease than men in KYH but similar odds as men in Tromsø 7. The between-study difference found in women was removed by additional adjustment for smoking history (odds ratio for association of study in women adjusted for age and pack-year history 1.02 95% CI 0.76, 1.37).

**Table 3:** Association Between Obstructive Lung Disease and Sex, Age, Education, Body Mass Index and Smoking by Study (Ages 40–69). Know Your Heart Study 2015–2018 and Tromsø Study 2015–2016

		KYH				Tromsø 7				Odds Ratio for Study KYH/Tromsø 7 Adjusted for Age and Sex	Likelihood Ratio Test for Interaction by Study Adjusted for Age and Sex
		Prevalence n/N (%)		Odds Ratio Age, Sex and City Adjusted Association with Risk Factor (95% CI)		Prevalence n/N (%)		Odds Ratio Age and Sex Adjusted Association with Risk Factor (95% CI)			
Total		163/1883	(8.7)			524/5217	(10.0)			0.89 (0.74, 1.07)	–
Sex	Men	88/787	(11.2)	1.00	(ref)	290/2831	(10.2)	1.00	(ref)	1.17 (0.90, 1.52)	P=0.002
	Women	75/1096	(6.8)	0.59	(0.43, 0.81)	234/2386	(9.8)	1.06	(0.88, 1.27)	0.69 (0.53, 0.90)	
Age, years	40–49	43/569	(7.6)	1.00	(ref)	99/1024	(9.7)	1.00	(ref)	0.77 (0.53, 1.12)	P=0.67
	50–59	53/643	(8.2)	1.10	(0.73, 1.68)	108/1242	(8.7)	0.88	(0.67, 1.27)	0.95 (0.67, 1.34)	
	60–69	67/671	(10.0)	1.35	(0.91, 2.02)	317/2951	(10.7)	1.13	(0.89, 1.43)	0.92 (0.70, 1.21)	
	Test for trend			P=0.13				P=0.15			
Education	Lower	19/121	(15.7)	1.65	(0.96, 2.85)	161/1197	(13.5)	1.55	(1.22, 1.99)	1.23 (0.73, 2.07)	P=0.13
	Middle	93/991	(9.4)	1.00	(ref)	135/1499	(9.0)	1.00	(ref)	1.12 (0.84, 1.48)	
	Higher	51/771	(6.6)	0.71	(0.49, 1.01)	220/2463	(8.9)	0.99	(0.79, 1.24)	0.73 (0.53, 1.00)	
	Test for trend			P=0.004				P<0.001			
Smoking status	Never	46/961	(4.8)	1.00	(ref)	93/1814	(5.1)	1.00	(ref)	0.92 (0.64, 1.34)	P=0.82
	Ex-smoker	38/458	(8.3)	1.73	(1.08, 2.77)	266/2584	(10.3)	2.10	(1.64, 2.68)	0.80 (0.56, 1.15)	
	Current smoker	78/462	(16.9)	4.14	(2.73, 6.29)	161/774	(20.8)	4.85	(3.70, 6.37)	0.86 (0.63, 1.18)	
Smoking Pack years (ever smokers)	Never smoked	46/961	(4.8)	1.00	(ref)	93/1814	(5.1)	1.00	(ref)	0.92 (0.64, 1.34)	P=0.90
	>0<10	21/269	(7.8)	1.80	(1.04, 3.10)	106/1504	(7.1)	1.39	(1.04, 1.86)	1.04 (0.63, 1.73)	
	10–19	16/158	(10.1)	2.59	(1.37, 4.87)	121/911	(13.3)	2.87	(2.16, 3.82)	0.92 (0.52, 1.65)	
	20–29	32/208	(15.4)	4.59	(2.66, 7.91)	92/466	(19.7)	4.64	(3.40, 6.34)	0.83 (0.51, 1.37)	
	30–39	23/123	(18.7)	5.62	(3.02, 10.47)	59/250	(23.6)	6.13	(4.25, 8.84)	0.82 (0.47, 1.43)	
	40+	22/98	(22.5)	7.55	(3.98, 14.32)	35/132	(26.5)	7.14	(4.56, 11.17)	0.70 (0.35, 1.41)	
	Test for trend			P<0.001				P<0.001			
Body mass index, kg/m^2^	<18.5	8/22	(36.4)	4.14 (1.65, 10.39)		11/31	(35.5)	3.67 (1.73, 7.78)		0.79 (0.18, 3.52)	P=0.60
	18.5–24.9	69/535	(12.9)	1.00 (ref)		222/1691	(13.1)	1.00 (ref)		1.15 (0.85, 1.55)	
	25–29.9	44/710	(6.2)	0.39 (0.26, 0.59)		203/2303	(8.8)	0.62 (0.51, 0.77)		0.70 (0.50, 0.99)	
	30–34.5	25/425	(5.9)	0.39 (0.24, 0.63)		60/901	(6.7)	0.46 (0.34, 0.62)		0.92 (0.56, 1.50)	
	>35	16/188	(8.5)	0.64 (0.36, 1.14)		26/284	(9.2)	0.66 (0.43, 1.02)		0.93 (0.47, 1.82)	
	Test for trend			P<0.001				P<0.001			
Self-reported CVD co-morbidity	No	150/1710	(8.8)	1.00 (ref)		478/4799	(10.0)	1.00 (ref)		0.92 (0.75, 1.12)	0.54
	Yes	13/167	(7.8)	0.73 (0.40, 1.33)		28/264	(10.6)	1.04 (0.69, 1.57)		0.72 (0.35, 1.46)	

The equivalent associations between obstructive lung function plus respiratory symptoms are shown in Table 4. In contrast to findings for obstructive lung function, there was strong evidence for higher odds of obstructive lung function plus symptoms in the KYH participants after adjustment for sex and age. This was seen in all ages and across categories of education and self-reported CVD morbidity. However, there was some evidence of effect modification by sex (p=0.06), pack years history (p=0.01) and smoking status (p=0.02) with largest between-study effect in never smokers and no evidence for a between-study effect in heavier smokers, and in women.

**Table 4:** Association Between Obstructive Lung Disease with Respiratory Symptoms (Cough and/or Breathlessness) and Sex, Age, Education, Body Mass Index and Smoking by Study (Ages 40–69). Know Your Heart Study 2015–2018 and Tromsø Study 2015–2016

		KYH				Tromsø 7				Odds Ratio for Study KYH/Tromsø 7 Adjusted for Age and Sex	Likelihood Ratio Test for Interaction by Study Adjusted for Age and Sex
		Prevalence n/N (%)		Odds Ratio Age, Sex and City Adjusted Association with Risk Factor (95% CI)		Prevalence n/N (%)		Odds Ratio Age and Sex Adjusted Association with Risk Factor (95% CI)			
Total		129/1844	(7.0)			272/5144	(5.3)			1.46 (1.17, 1.83)	
Sex	Men	65/770	(8.4)	1.00	(ref)	121/2347	(5.1)	1.00	(ref)	1.78 (1.30, 2.46)	P=0.06
	Women	64/1074	(6.0)	0.69	(0.48, 0.99)	151/2797	(5.4)	1.06	(0.83, 1.35)	1.23 (0.91, 1.68)	
Age, years	40–49	31/559	(5.5)	1.00	(ref)	37/1015	(3.7)	1.00	(ref)	1.58 (0.97, 2.57)	P=0.84
	50–59	44/629	(7.0)	1.29	(0.80, 2.07)	56/1224	(4.6)	1.26	(0.83, 1.93)	1.58 (1.05, 2.37)	
	60–69	54/656	(8.2)	1.52	(0.96, 2.40)	179/2905	(6.2)	1.74	(1.21, 2.49)	1.36 (0.99, 1.87)	
	Test for trend			P=0.07				P<0.001			
Education	Lower	15/117	(12.8)	1.64	(0.90. 3.00)	119/1170	(10.2)	2.17	(1.59, 2.95)	1.34 (0.75, 2.38)	P=0.67
	Middle	74/968	(7.6)	1.00	(ref)	71/1483	(4.8)	1.00	(ref)	1.79 (1.27, 2.52)	
	Higher	40/759	(5.3)	0.69	(0.46, 1.03)	76/2437	(3.1)	0.65	(0.47, 0.91)	1.76 (1.18, 2.62)	
	Test for trend			P=0.007				P<0.001			
Smoking status	Never	37/943	(3.9)	1.50	(0.87, 2.59)	27/1800	(1.5)	1.00	(ref)	2.50 (1.49, 4.21)	P=0.02
	Ex-smoker	25/451	(5.5)	1.00	(ref)	138/2563	(5.4)	3.55	(2.33, 5.39)	1.19 (0.75, 1.87)	
	Current smoker	67/450	(14.9)	4.70	(2.98, 7.42)	107/764	(14.0)	10.52	(6.83, 16.20)	1.18 (0.83, 1.67)	
Smoking Pack years	Never smoked	37/943	(3.9)	1.00	(ref)	27/1800	(1.5)	1.00	(ref)	2.50 (1.49, 4.21)	P=0.01
	>0<10	18/264	(6.8)	1.98	(1.10, 3.56)	41/1491	(2.8)	1.80	(1.10, 2.94)	2.89 (1.57, 5.30)	
	10–19	13/157	(8.3)	2.88	(1.44, 5.78)	65/898	(7.2)	5.09	(3.22, 8.04)	1.46 (0.75, 2.83)	
	20–29	23/200	(11.5)	4.44	(2.40, 8.21)	64/464	(13.8)	10.31	(6.47, 16.43)	1.01 (0.57, 1.79)	
	30–39	17/120	(14.2)	5.37	(2.67, 10.81)	41/250	(16.4)	13.36	(7.97, 22.39)	0.95 (0.50, 1.80)	
	40+	19/98	(19.4)	8.45	(4.22, 16.90)	28/131	(21.4)	18.40	(10.34, 32.75)	0.66 (0.31, 1.40)	
	Test for trend			P<0.001				P<0.001			
Body mass index, kg/m^2^	<18.5	5/21	(23.8)	3.00 (1.04, 8.64)		7/31	(22.6)	4.78 (2.00, 11.46)		0.94 (0.16, 5.45)	P=0.14
	18.5–24.9	53/525	(10.1)	1.00 (ref)		95/1672	(5.7)	1.00 (ref)		2.55 (1.75, 3.72)	
	25–29.9	38/693	(5.5)	0.45 (0.29, 0.71)		110/2267	(4.9)	0.82 (0.62, 1.10)		1.19 (0.81, 1.75)	
	30–34.5	21/420	(5.0)	0.42 (0.25, 0.71)		39/887	(4.4)	0.74 (0.51, 1.10)		1.20 (0.69, 2.10)	
	>35	11/183	(6.0)	0.54 (0.27, 1.07)		19/280	(6.8)	1.24 (0.74, 2.06)		0.87 (0.39, 1.94)	
	Test for trend			p<0.001				p=0.22			
Self-reported CVD co-morbidity	No	115/1677	(6.9)	1.00 (ref)		242/4734	(5.1)	1.00 (ref)		1.52 (1.20, 1.93)	P=0.74
	Yes	13/161	(8.1)	0.99 (0.54, 1.83)		17/258	(6.6)	1.19 (0.71, 1.99)		1.27 (0.58, 2.76)	

In sensitivity analysis using the stricter definition of respiratory symptoms (Supplementary Table 1), the between study difference was larger. There remained evidence for effect modification by pack-years smoked (no between study difference in heavier smokers >20 pack-years) but there was no evidence for effect modification by the other risk factors.

Observed interactions between age and pack-year history within each study are shown in Supplementary Table 2. There was some evidence for interaction between age and pack-year history after adjusting for sex for the outcome obstructive lung function for Tromsø 7 with stronger association between pack-year history and age in the older participants (p=0.06). Conversely, there was good evidence for interaction between age and smoking pack-year history for obstructive lung function plus one of more respiratory symptoms but with stronger association between pack-year history and the outcome in the younger age groups (p=0.01). There was no evidence for interactions between age and pack-year history with obstructive lung function for KYH (p=0.18) or obstructive lung function plus 1 or more symptoms in Tromsø 7 (p=0.46).

### Awareness and Management Among Those with Obstructive Lung Function

The levels of awareness and management among those with obstructive lung function by study, sex and reported respiratory symptoms are shown in Table 5. There was evidence for a difference in the between-study association with current smoking by sex therefore associations for current smoking are shown stratified by sex. There was no evidence for an interaction between sex and study for levels of awareness (test for interaction p=0.44) and pharmacological managements (maintenance treatment test for interaction p=0.37; relief of symptoms p=0.91).

**Table 5:** Awareness and Management by Study and Level of Self-Reported Respiratory Symptoms in Those with Obstructive Lung Function. Know Your Heart Study 2015–2018 and Tromsø Study 2015–2016

		No Symptoms (n=29 KYH) (n=246 Tromsø 7)		Cough or Breathless (n=58 KYH) (n=196 Tromsø 7)		Cough and Breathless (n=71 KYH) (n=76 Tromsø 7)		Test for Trend with Symptoms (Adjusted Age and Sex)	Total (n=158 KYH*) (n=493 Tromsø 7)	
		N	(%)	N	(%)	N	(%)		N	(%)
Self-report ever diagnosed	KYH	2	(6.9)	14	(24.1)	41	(57.8)	P<0.001	57	(36.1)
	Tromsø 7	11	(4.6)	39	(21.2)	33	(48.5)	P<0.001	83	(16.8)
	Age and sex adjusted OR Tromsø 7/KYH (95% CI)	0.48	(0.09, 2.44)	0.70	(0.34, 1.45)	0.59	(0.29, 1.20)		0.29	(0.19, 0.44)
Currently smokes	KYH men	6	(33.3)	15	(53.6)	28	(75.7)	P=0.003	49	(59.0)
	Tromsø 7 men	19	(17.3)	30	(38.0)	24	(57.1)	P<0.001	73	(31.6)
	Age adjusted OR Tromsø 7/KYH	0.43	(0.13, 1.38)	0.54	(0.22, 1.36)	0.41	(0.14, 1.23)		0.30	(0.18, 0.51)
	KYH women	2	(18.2)	9	(30.0)	15	(44.1)	P=0.03	26	(34.7)
	Tromsø 7 women	33	(24.6)	36	(30.8)	17	(50.0)	P=0.008	86	(30.2)
	Age adjusted OR Tromsø 7/KYH	1.00	(0.19, 5.26)	1.13	(0.45, 2.85)	1.71	(0.52, 5.59)		0.87	(0.50, 1.51)
Maintenance treatment^a^	KYH	1	(3.7)	4	(7.3)	9	(12.9)	P=0.12	14	(9.2)
	Tromsø 7	17	(6.9)	31	(15.8)	28	(36.8)	P<0.001	76	(14.7)
	Age and sex adjusted OR Tromsø 7/KYH	1.52	(0.18, 12.77)	2.46	(0.80, 7.56)	3.80	(1.50, 9.63)		1.56	(0.85, 2.88)
Short acting symptomatic treatment^b^	KYH	0	(0.0)	2	(3.6)	5	(7.1)	P=0.12	7	(4.6)
	Tromsø 7	6	(2.4)	17	(8.7)	15	(19.7)	P<0.001	38	(7.3)
	Age and sex adjusted OR Tromsø 7/KYH	-		3.18	(0.68, 14.88)	4.49	(1.19, 16.97)		1.72	(0.74, 3.98)

**Notes:** *Data on medication use missing in Know Your Heart for 6 participants with obstructive lung disease (1 participant with symptoms). ^a^ATC codes R03BB, A03AC12, 13, 18, 19, R03AK, R03AL, R03DA04, R03DX07. ^b^ATC codes R03AC02, 03, 04.

### Awareness of COPD

There was strong evidence that awareness of COPD was lower among the Tromsø 7 participants (age and sex adjusted odds ratio 0.29 (95% CI 0.19, 0.44). However, awareness was strongly related to reporting of respiratory symptoms (Table 5) and on adjustment for respiratory symptoms this association was substantially reduced (OR 0.62 (95% CI 0.38, 1.01).

### Smoking and Smoking Cessation

After adjusting for age, current smoking among those with obstructive lung function was more common among the KYH participants in men (OR 3.30 95% CI 1.95, 5.61) but similar in KYH/Tromsø 7 participants in women (OR 1.15 95% CI 0.66, 2.01). In both studies, the prevalence of being a current smoker was higher among participants reporting respiratory symptoms (Table 5).

Among the KYH participants who had ever smoked the majority of those with obstructive lung disease plus symptoms had been advised to stop smoking by a doctor (62/90 68.9%). However, the proportion of these participants who reported they were offered assistance to stop smoking was much lower (7/90 8%).

### Pharmacological Management

The prevalence of use of medications for COPD by study and reporting of respiratory symptoms is shown in Table 5. The majority of participants with obstructive lung function were not using medications for management of COPD. The use of medications for maintenance and for symptom relief was higher in those reporting respiratory symptoms in Tromsø 7 (test for trend p<0.001) but there was only weak evidence for an association between medication use and symptoms in KYH (test for trend p=0.12) although the numbers reporting any medication use were extremely low limiting power to detect any association. While there was no evidence for a difference in medication use between studies after adjusting for age and sex (Table 5), after additional adjustment for the level of reported symptoms there was strong evidence that the odds of receiving maintenance therapy (OR 2.90 95% CI 1.48, 5.70) and short-acting treatments (OR 3.56 95% CI 1.43, 8.87) for symptoms relief were higher for participants in Tromsø 7.

### Impact of Missing Data on Spirometry Testing

The characteristics of those who did and did not complete the spirometry examination in both studies are shown in Supplementary Table 3. The factors associated with completing spirometry differed between the two studies. In KYH the main factor associated with spirometry was age with good evidence that those with spirometry were younger than all participants attending the health check. There was also weak evidence those with spirometry were more highly educated, had lower BMI and reported less breathlessness. In Tromsø 7, there were substantial differences in age between those with and without spirometry but in contrast to KYH those with spirometry data were older in keeping with additional inclusion of those who attended previous examinations in the sample selection. There was also evidence that those with spirometry were more likely to be women, have lower levels of education, be ex-smokers, have lower BMI, and less likely to report existing CVD.

The prevalence of obstructive lung disease estimated in sensitivity analyses using multiple imputation by age and sex is shown in Supplementary Figures 2A and B. The substantive findings were not different using multiple imputation to complete case analysis.

## Discussion

In this study comparing prevalence of obstructive lung function between participants aged 40–69 years taking part in population-based studies in Russia and Norway we found no evidence for a difference in obstructive lung function in men, but higher prevalence of obstructive lung function in the Norwegian compared to Russian women, which was explained by differences in smoking history. In contrast, the prevalence of COPD defined as both obstructive lung function and respiratory symptoms was higher among both men and women in the Russian study. There was a strikingly high prevalence of respiratory symptoms among Russian participants both among those who had an obstructive lung function pattern on spirometry but also in participants without obstructive lung function, reflecting very different patterns of symptom reporting in the two populations.

The age-standardized prevalence of obstructive lung function (pre-bronchodilator) among the Russian participants was 11.0% using the GLI-LLN normal definition in men and 6.8% in women. This is higher than the findings from pre-bronchodilator spirometry tests reported by Andreeva et al in the RESPECT study in North-West Russia (9.6% in men 4.8% in women)^13^ and the Ural (5.8% total population).^15^ The findings among the Tromsø Study participants were also higher than estimates from the HUNT study from central Norway in 2006–2008 (7.3%).^19^ Prevalences using a fixed cut point rather than LLN were more similar to a previous study from Novosibirsk from 2002–5 which found 19.5% (23.5% in men and 16.0% in women) using a broader definition of airway obstruction on spirometry (FEV_1_/FVC ratio <0.7 or FEV_1,_<80%).^14^ In this study, we did not find any evidence for a difference in the prevalence of obstructive lung function between men in the Russian and Norwegian studies. This is surprising given historically very high smoking prevalence among Russian men.^7^ Despite recent declines in smoking in Russia,^28^ we did find here that the prevalence of current smoking and pack-year history was higher among the Russian than the Norwegian men. However, there was also a high prevalence of ex-smokers in the Norwegian sample therefore the current lung function damage in this sample could be attributable to higher levels of smoking in Norway in the past. There was no statistical evidence for a higher burden of airway obstruction in the Russian men, although the actual prevalence was slightly higher in the older ages group (60–69 years). The lower than anticipated estimates of obstructive lung function in Russian men found here and in previous studies are difficult to interpret but given the very high premature CVD mortality in Russian men in this age range, differential survival may play a role. There was no evidence for an association with COPD and self-report of MI or stroke in this study in keeping with this, however it is important to note our measure was based on self-reported disease only which could have been affected by measurement error, there was a small number of cases and we did not include a detailed investigation of the relationships with all CVD outcomes. The high burden of CVD mortality in Russia makes investigation of cardiovascular and respiratory co-morbidity in this population an important area to investigate in more depth. Prospective studies to investigate the incidence of COPD are also needed.

In contrast to findings from spirometry testing, there were striking differences between the Russian and Norwegian participants with regards to reporting of symptoms. The high burden of respiratory symptoms in the Russian study population is important given increasing evidence that respiratory symptoms among smokers are associated with poorer outcomes including a higher rate of respiratory infections, impaired exercise capacity, airway thickening, and poorer quality of life even in the absence of obstructive lung function on spirometry.^29^,^30^ In a prospective study of 596 smokers and former smokers aged 70–79 years mortality was similar in those with dyspnoea but no obstructive lung function compared to those with obstructive lung function without dyspnoea.^31^ Several previous studies have found the prevalence of reported respiratory symptoms is very high in Russia consistent with the levels of breathlessness and chronic cough found here.^10–13^ Only two of these studies also included findings from spirometry. In the study by Chuchalin et al spirometry was only conducted in those who reported either respiratory symptoms or risk factors, of whom 21.8% also had airway obstruction.^11^ Andreeva et al reported on data from both lung function testing and respiratory symptoms and found that the positive predictive value of respiratory symptoms for identifying obstructive lung function was low (8%)^13^ which was similar to findings in this study in both Russian (10%) and Norwegian participants (14%). Here we also found a high prevalence of respiratory symptoms in the Russian study population in those without obstructive lung function suggesting there are other explanations aside from COPD per se for high burden of respiratory symptoms found here and in previous studies in Russia. The symptoms considered here (cough and breathlessness) are non-specific and may be caused by many factors including both other respiratory diseases (for instance lower respiratory tract infection was the 6th and tuberculosis the 18th leading cause of death in Russia in 2016^1^) and non-respiratory causes. For example, the differences in levels of breathlessness in the population may be related to anxiety, physical fitness, levels of obesity or other co-morbidities in particular heart failure. Differences in air pollution may also play an important role as well as possible cultural differences in the interpretation or perception of symptoms. The lower levels of use of medications found here among the Russian participants with obstructive lung function could also be a factor with differences in management influencing levels of symptom control. Due to the cross-sectional nature of the data, we could not investigate this hypothesis here due to strong possibility of confounding by indication (those with symptoms were more likely to receive medication due to increased need).

The presence of respiratory symptoms in those with obstructive lung function was important when comparing awareness and management of COPD between the two studies. Among those with obstructive lung disease, respiratory symptoms were associated in a dose-response manner with higher awareness of disease, smoking and among the Norwegian participants the use of medications for management of COPD. The relatively large asymptomatic group of Norwegian participants with obstructive lung disease as defined by spirometry were less likely to report a diagnosis or any pharmacological treatment. However, higher levels of awareness among the Russian participants did not translate into correspondingly better pharmacological management as levels of pharmacological treatment were low while many participants continued to smoke. Smoking cessation is a key part of the management of COPD. The prevalence of smoking in those with obstructive lung function was particularly high in the Russian men (59% current smokers) compared to approximately 30% in the Norwegian participants and Russian women. In the KYH study, some additional questions about smoking cessation were asked to smokers. While the majority of the Russian participants who had both obstructive lung function and respiratory symptoms had been advised by a doctor to stop smoking, only 8% of this high-risk group reported they had been offered assistance to stop. Increasing the availability of smoking cessation treatments could have a substantial benefit in reducing the burden from COPD and other smoking-related disease.

This study has several limitations which should be considered on interpreting the findings:

First, here we have estimated prevalence of obstructive lung function (with respiratory symptoms) in participants in population-based studies. It is likely the findings may have been affected by selection bias. While we investigated the potential impact on the findings of using a sub-set of participants and found no evidence that this had a substantial impact of the prevalence of obstructive lung disease, there may still be bias in the extent to which participants are representative of their respective populations. The proportion of invited participants who took part in the studies overall was 22% for Novosibirsk, 60% for Arkhangelsk^21^ and 65% for Tromsø 7.^32^ It is plausible attendance was differential by lung function status, as those with very severe respiratory disease may be less likely to take part. Any selection bias will have affected the prevalence estimates reported here and these should be interpreted with caution. Furthermore, the studies took place in two cities in Russia and one municipality in Norway, therefore prevalence estimates may not be generalizable to the whole of both countries. However, comparisons of use of COPD medication as maintenance treatment within the Norwegian Prescription Database (NorPD) show that use in the Troms and Finnmark county was very close to Norway as a whole in 2016.^33^

Secondly, in both studies only pre-bronchodilator spirometry was conducted while for a diagnosis of COPD spirometry should also be conducted post-bronchodilator in order to demonstrate irreversible airflow limitation, therefore some participants with reversible airflow limitation may have been misclassified. Sputum production was also not assessed in either study. The two studies used different spirometry devices which may have limited the comparability of the results although protocols for data collection were similar and procedures for quality control were harmonised. The questions on chronic cough were also not identical although the questions on breathlessness in both studies were measured using the same standardized tool. Translation of the questions on breathlessness to Russian and Norwegian were cross-checked by a speaker of both languages and found to be consistent in meaning. While measurement error due to differences in how questions on respiratory symptoms were asked is possible it does not seem sufficient to account for the huge differences in reporting of symptoms observed in this study. However, cultural differences in the perception of symptoms for example understanding of the concept of breathlessness may play a role in accounting for the large population level differences observed in this study. Finally, we were restricted in assessing management to pharmacological management and have not been able to compare other non-pharmacological aspects of COPD management such as participation in pulmonary rehabilitation programmes.

In conclusion, we have found that the burden of obstructive lung disease on spirometry was similar in participants taking part in population-based studies in Norway and Russia but there was a strikingly high burden of respiratory symptoms among the Russian participants. Further work is needed to understand the reasons and implications for health of this high prevalence of chronic cough and breathlessness. The contribution of cardiovascular disease, in particularly heart failure, here as well as further understanding of the burden of respiratory and cardiovascular co-morbidity are important areas to investigate. There were low levels of smoking cessation and use of medications in both study populations in participants identified with COPD indicating management of COPD could be improved in both countries.

## Data Sharing Statement

The data that support the findings of this study are available from Know Your Heart and The Tromsø Study, but restrictions apply to the availability of these data, which were used under license for the current study, and so are not publicly available. Data from the Know Your Heart Study are however available upon reasonable request to the Know Your Heart Study.^34^ For The Tromsø Study, data are available subject to scientific and ethical approval of a study protocol.^32^
